# Genetically Predicted Circulating Omega-3 Fatty Acids Levels Are Causally Associated With Increased Risk for Systemic Lupus Erythematosus

**DOI:** 10.3389/fnut.2022.783338

**Published:** 2022-02-09

**Authors:** Peng Wang, Kun Xiang, Yuan-Yuan Xu, Yi-Sheng He, Yu-Qian Hu, Jing Ni, Hai-Feng Pan

**Affiliations:** ^1^Teaching Center for Preventive Medicine, School of Public Health, Anhui Medical University, Hefei, China; ^2^Inflammation and Immune Mediated Diseases Laboratory of Anhui Province, Hefei, China; ^3^Department of Epidemiology and Biostatistics, School of Public Health, Anhui Medical University, Hefei, China; ^4^Department of Outpatient Wound Care Center, 901 Hospital of Joint Logistics Support Force of People Liberation Army, Hefei, China

**Keywords:** omega-3 fatty acids, omega-6 fatty acids, systemic lupus erythematosus, SLE, Mendelian randomization

## Abstract

**Background:**

Accumulating evidence has demonstrated the associations of omega-3 or omega-6 polyunsaturated fatty acids (PUFAs) with the disease activity and inflammatory mediators of systemic lupus erythematosus (SLE), but the evidence of causal links of omega-3 or omega-6 PUFAs on the risk for SLE remains inconclusive.

**Objectives:**

This study was conducted to evaluate the causal relationships between omega-3/omega-6 PUFAs and SLE by performing the Mendelian randomization (MR) analysis.

**Methods:**

Genome-wide significant single-nucleotide polymorphisms (SNPs) were obtained from genome-wide association studies (GWASs) of circulating omega-3/omega-6 levels (*n* = up to 13,544) and GWAS meta-analyses of SLE (*n* = 14,267), respectively. The bidirectional two-sample MR (TSMR) analysis was conducted to infer the causality.

**Results:**

The inverse-variance weighted (IVW) method revealed that genetically determined per SD increase in omega-3 levels were causally associated with an increased risk for SLE (odds ratios [*OR*s] = 1.49, 95% *CI*: 1.07, 2.08, *p* = 0.021), but no causal effect of omega-6 on the risk SLE was observed (IVW *OR* = 1.06, 95% *CI*: 0.72, 1.57, *p* = 0.759). In addition, there were no significantly causal associations in genetic predisposition to SLE with the changes of omega-3 and omega-6 levels, respectively (IVW beta for omega-3: 0.007, 95% *CI*: −0.006, 0.022, *p* = 0.299; IVW beta for omega-6: −0.008, 95% *CI*: −0.023, 0.006, *p* = 0.255).

**Conclusion:**

The present study revealed the possible causal role of omega-3 on increasing the risk for SLE, it could be the potential implications for dietary recommendations.

## Introduction

Systemic lupus erythematosus (SLE) is an autoimmune inflammatory connective tissue disease involving multiple organs and tissues that are characterized as the loss of immune tolerance and immune-complex deposition (1, 2). Increasing evidence has demonstrated that the disease onset of SLE was triggered by the interactions of genetic susceptibility and several environmental factors (3–5). Up to date, the associations of genetic components with SLE have been extensively studied, and there were more than one hundred susceptibility loci for SLE that have been identified (6, 7). Despite the implementation of many large-scale genome wide association studies (GWASs) with SLE, a better understanding of the causal roles of genetic susceptible loci would be helpful for the treatment and prevention of SLE.

Nutrition is an environmental factor of major importance. Polyunsaturated fatty acids (PUFAs) are a type of essential fatty acids that cannot be synthesized by humans. It has been demonstrated that PUFAs are localized in cell membrane and involve in a large number of physiological functions, such as inflammation, blood sugar control, regulation of blood pressure, and cell signaling (8–11). Based on the different position of the first double bond, PUFAs could be classified into two major classes: omega-3 and omega-6. Evidence from human and animal studies indicated that omega-3 PUFAs, especially eicosapentaenoic acid (EPA) and docosahexaenoic acid (DHA), have anti-inflammation properties that showed some improvements in the rheumatic diseases (12–14). Recent meta-analysis of five randomized controlled trials (RCTs) suggested that the daily supplementation of omega-3 was more effective in alleviating the disease activity of SLE as compared with placebo (15). In contrast to omega-3, daily omega-6-rich diet has been shown to increase the levels of autoantibodies and causes proteinuria and glomerulonephritis in New Zealand black/New Zealand white (NZB/W) F1 mouse model of spontaneous SLE (16). Moreover, each unit increase of the omega-6 to omega-3 ratio was associated with an increased systemic lupus activity questionnaire (SLAQ) point (17). Although the emerging roles of omega-3 and omega-6 PUFAs in the treatment of SLE have been proposed, the causal associations of these two PUFAs with SLE are still unclear.

Mendelian randomization (MR) analysis is an emerging epidemiological technique that uses genetic variations as natural instrumental variables (IVs) to infer the causal correlation of exposure factors on health outcomes (18–22). MR relies on the natural, random assortment of genetic variants during meiosis yielding a random distribution of genetic variants in the population, thus MR, analogous to RCTs, is less prone to confounding and reverse causation than traditional observational studies (23). Two-sample MR analysis (TSMR) is a novel extension of MR, as compared with one-sample MR (effect estimates derived from the same sample), TSMR extracts the genetic effect estimates from two non-overlapping sets of individuals that would be effective to strengthen the causal inference (24).

In the present study, we obtained the genetic summary data from two independent large GWASs, and conducted the bidirectional TSMR analysis to infer the causal associations of omega-3 and omega-6 with SLE.

## Materials and Methods

**Figure 1:**
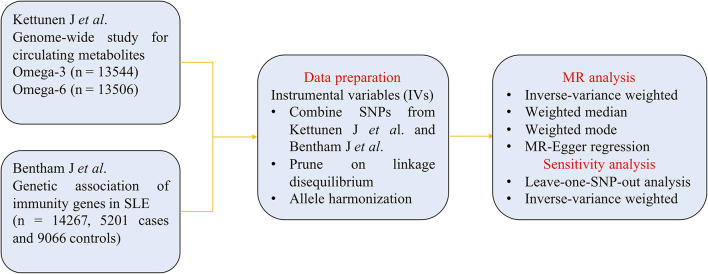
The flowchart of Mendelian randomization (MR).

The flowchart of MR analysis is displayed in Figure 1. We obtained genetic summary data from two large GWASs, after data prune and allele harmonization, MR analyses with four methods and sensitivity analyses were conducted to infer the causal associations between omega-3/omega-6 and SLE.

### Study Design

To minimize the distortion effects of confounding factors, the genetic variants used as IVs in MR analysis should meet three key assumptions: (1) the selected IVs must be strongly associated with the risk factors of interest (omega-3 or omega-6). In this study, *F* statistic was used to confirm the correlation strength between instrumental variables and exposure. *F* is expressed as *R*^2^ (*n*-*k*-1)/[*k* (1-*R*^2^)]. *R*^2^ represents the cumulative explained variance of the included single-nucleotide polymorphisms (SNPs) at the circulating omega-3 or omega-6 levels, *n* refers to the sample size, and *k* is the number of included SNPs. When *F* > 10, the correlation is considered strong enough to avoid the deviation caused by weak IVs (25); (2) IVs are independent of confounding factors; (3) IVs can only influence the outcome through their effect on exposure, nor are causal pathway to outcome other than exposure. MR-Egger regression was used to confirm the horizontal pleiotropy pathway between IVs and outcome (26).

### Data Sources

In the current study, effect estimates of omega-3 and omega-6 associated SNPs were obtained from the published GWAS associations of the Kettunen J et al.'s consortium, which included up to 13,544 European ancestry (27). In their study, omega-3 and omega-6 were defined as circulating metabolites in the peripheral blood. Summary statistics of omega-3 and omega-6 related SNPs with genome-wide significance (*p* < 5 × 10^−8^) were collected as candidate IVs. The linkage disequilibrium (LD) among selected SNPs was tested within the condition of *r*^2^ < 0.001 to minimize the impact of strong LD. After clumping algorithm, there were 5 omega-3 SNPs and 9 omega-6 SNPs that were allocated for exposure datasets. The detailed information regarding effect allele (EA), other allele, effect allele frequency (EAF), effect sizes (Beta), SEs and *p* for omega-3 and omega-6 are displayed in Supplementary Tables 1, 2, respectively.

Genetic associations of SLE (outcome) were retrieved from another largest public GWAS meta-analysis of Bentham J et al., which included the genotyped dataset of 14,267 study subjects (5,201 patients with SLE and 9,066 controls) (28). The corresponding genetic information of SNPs about omega-3 and omega-6 were reviewed and collected in SLE consortium, respectively (Supplementary Tables 3, 4).

### Statistical Analysis

Summary statistics about the exposure and outcome datasets were harmonized to maintain the effect of allele always reflecting the same allele between two datasets. TSMR analyses with inverse-variance weighted (IVW), median-based estimator (weighted median and weighted mode), and MR-Egger regression methods were implemented to infer the causality. The method of IVW equates to conduct a weighted linear regression of the correlation of the IVs with the outcome (29). It requires that all IVs are valid and without pleiotropic effects. Median-based estimator is used to combine multiple genetic variations into a causal estimate and it is suitable for the situations where up to 50% of the information comes from invalid IVs (30). MR-Egger regression method is used to confirm whether there are horizontal pleiotropic effects existence and its slope represents the potential causal effect (31). The heterogeneity between individual genetic variant was calculated, and leave-one-out sensitivity analysis was performed to test the stability and reliability of the pooled effect sizes of causal inference. All statistical analyses were performed in R (version 3.6.20 using the TSMR R package). Statistical significance was set as two-tailed *p* < 0.05.

## Results

### Selection of IVs

Kettunen J et al. have identified a number of genome-wide significant SNPs that were associated with the circulating omega-3 (8 SNPs) and omega-6 (12 SNPs) levels. However, six genome-wide significant SNPs were excluded due to the following reasons: LD with other SNPs (omega-3: rs28834423 and rs28361029; omega-6: rs190934192), being palindromic (omega-6: rs10402112), and no corresponding SNPs in SLE consortium (omega-3: rs12524498; omega-6: rs76246956). Thus, the remaining five omega-3 SNPs and nine omega-6 SNPs that were selected as IVs and included for TSMR analysis, respectively. The five SNPs for omega-3 explained about 2.5% of the variances in circulating omega-3 levels. While, the nine SNPs for omega-6 explained the variances in circulating omega-6 levels were about 4.8%. In addition, to exclude the potential impacts of weak IVs, we used *F* statistic to test the correlation strength of IVs with exposure, and no evidence of significant weak IVs among the selected SNPs were observed (all *F* > 10).

### Causal Effects of Omega-3/Omega-6 on SLE

**Figure 2:**
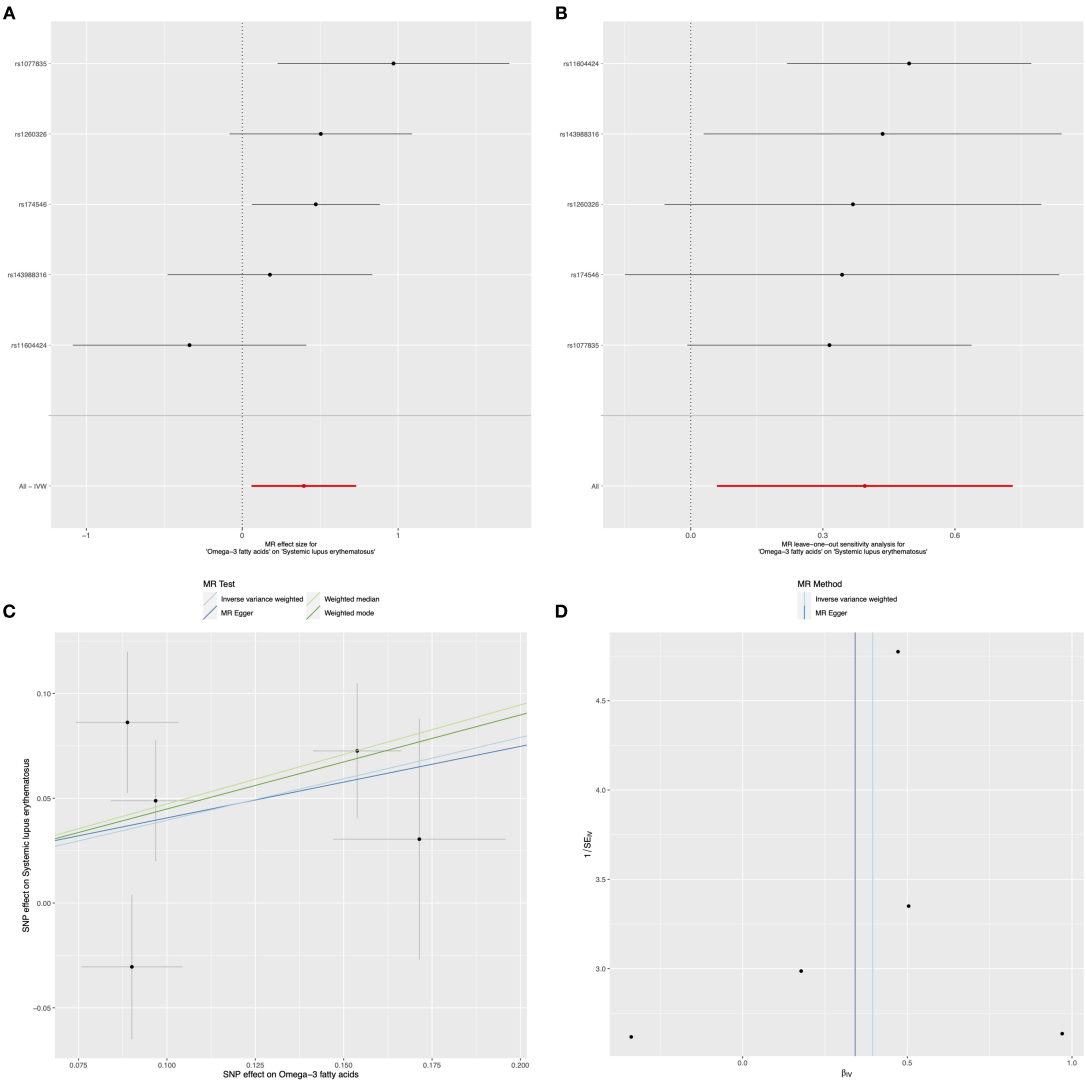
Mendelian randomization study of the effects of omega-3 levels on systemic lupus erythematosus (SLE). Forest plot **(A)**, leave-one-out sensitivity analysis **(B)**, scatter plot **(C)**, and funnel plot **(D)** of the effect of circulating omega-3 levels on SLE.

The results of IVW method indicated that genetically determined per SD increase in omega-3 showed positive association with disease risk for SLE, with the odds ratio (*OR*) of 1.49 (95% *CI*: 1.07, 2.08, *p* = 0.021) (Figure 2A and Table 1), the weighted median method yielded the consistent results (*OR* = 1.61, 95% *CI*: 1.14, 2.26, *p* = 0.007) (Table 1). Sensitivity analyses with the leave-one-out method suggested that such associations were not driven by any SNPs (Figure 2B). The horizontal pleiotropy of selected five SNPs on SLE was assessed by MR-Egger regression, and the results showed no evidence of horizontal pleiotropy in our analysis (intercept = 0.006; SE = 0.088, *p* = 0.946) (Figure 2C and Table 1). Furthermore, MR-Egger and IVW methods unveiled that there was no significant heterogeneity among the selected five SNPs in the causal inference between omega-3 and SLE (MR-Egger *p* = 0.084 and IVW *p* = 0.154) (Figure 2D and Table 1).

**Table 1:** Causal effects of Omega-3 on the risk for systemic lupus erythematosus (SLE).

**Exposure/Outcome**	**Methods**	**SNP (n)**	**OR**	**95% CI**		***P* value**
Omega-3 fatty acids/SLE	MR-Egger	5	1.41	0.32	6.12	0.681
	IVW	5	1.49	1.07	2.08	**0.021**
	Weighted median	5	1.61	1.14	2.26	**0.007**
	Weighted mode	5	1.57	1.07	2.30	0.083

*Test for Heterogeneity: P = 0.084 (MR-Egger) and P = 0.154 (IVW)*.

*Test for Horizontal pleiotropy: MR-Egger intercept = 0.006, se = 0.088, P = 0.946*.

*OR, odds ratio; IVW, inverse-variance weighted; SLE, systemic lupus erythematosus*.

**Figure 3:**
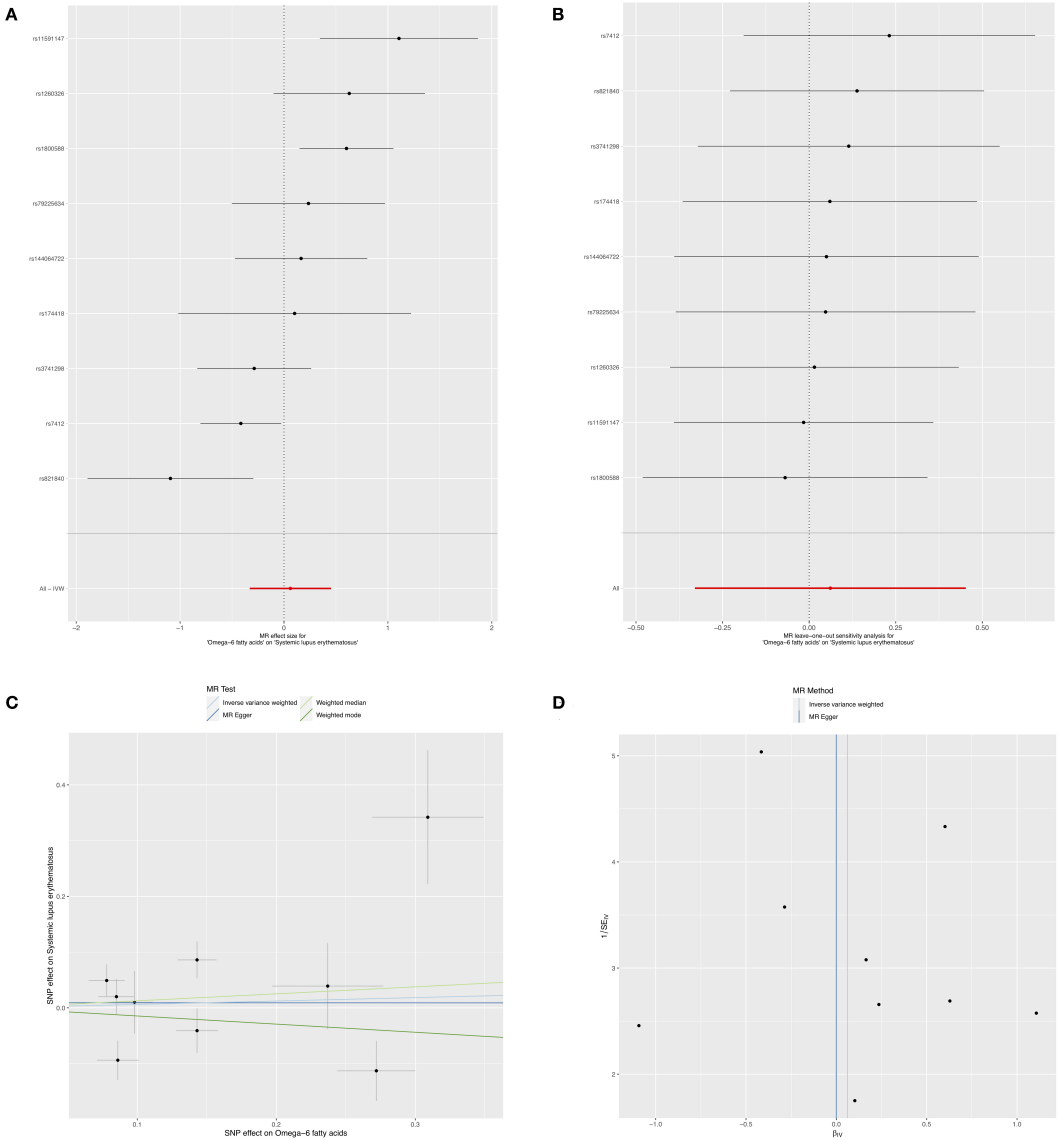
Mendelian randomization study of the effects of omega-6 levels on SLE. Forest plot **(A)**, leave-one-out sensitivity analysis **(B)**, scatter plot **(C)**, and funnel plot **(D)** of the effect of circulating omega-6 levels on SLE.

In relation to the causal effects of omega-6 on SLE, both the IVW and weighted median methods found that the changes of circulating omega-6 levels were not causally associated with the risk for SLE (IVW: *OR* = 1.06, 95% *CI*: 0.72, 1.57, *p* = 0.759; weighted median: *OR* = 1.13, 95% *CI*: 0.80, 1.61, *p* = 0.485) (Figure 3A and Table 2). Further sensitivity analysis reported the consistent results that omega-6 was not causally linked to SLE (Figure 3B). MR-Egger regression revealed that there was no horizontal pleiotropy among the selected nine SNPs (intercept = 0.009; SE = 0.066, *p* = 0.893) (Figure 3C and Table 2). However, the marked heterogeneity across the selected nine SNPs were observed (MR-Egger *p* = 0.001 and IVW *p* = 0.001) (Figure 3D and Table 2).

**Table 2:** Causal effects of Omega-6 on the risk for SLE.

**Exposure/Outcome**	**Methods**	**SNP (n)**	**OR**	**95% CI**		***P* value**
Omega-6 fatty acids/SLE	MR-Egger	9	1.00	0.38	2.63	0.999
	IVW	9	1.06	0.72	1.57	0.759
	Weighted median	9	1.13	0.80	1.61	0.485
	Weighted mode	9	0.86	0.50	1.49	0.614

*Test for Heterogeneity: P = 0.001 (MR-Egger) and P = 0.001 (IVW)*.

*Test for Horizontal pleiotropy: MR-Egger intercept = 0.009, se = 0.066, P = 0.893*.

*OR, odds ratio; IVW, inverse-variance weighted; SLE, systemic lupus erythematosus. The bold values indicate statistical significance with P-value < 0.05*.

### Causal Effects of SLE on Omega-3/Omega-6

**Figure 4:**
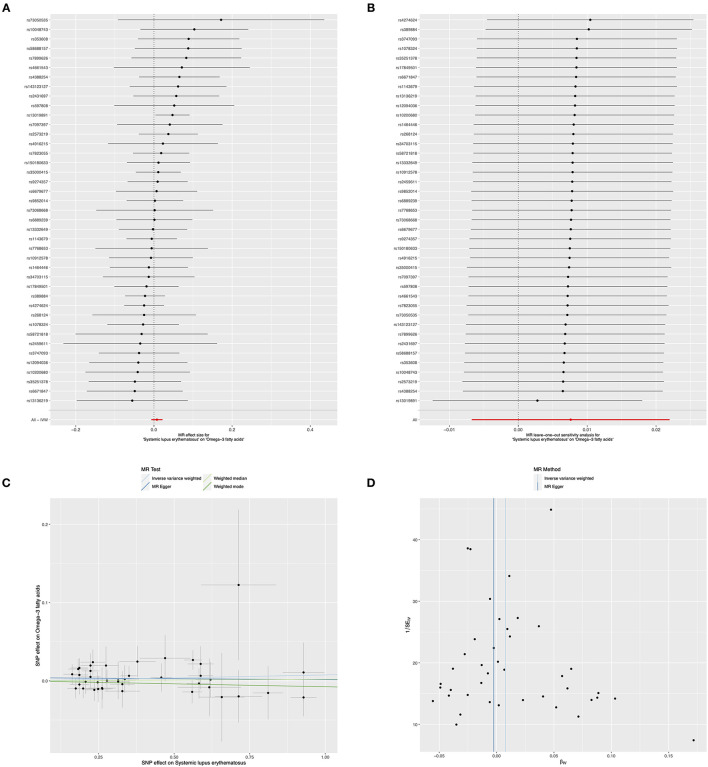
Mendelian randomization study of the effects of SLE on omega-3 levels. Forest plot **(A)**, leave-one-out sensitivity analysis **(B)**, scatter plot **(C)**, and funnel plot **(D)** of the genetically risk of SLE on circulating omega-3 levels.

In contrast, to investigate the causal effects of SLE on circulating omega-3, we set SLE as exposure and circulating omega-3 as outcome to infer the causality. There was no evidence of a significant relationship between SLE and circulating omega-3 levels, with the IVW beta of 0.007 (95% *CI*: −0.006, 0.022, *p* = 0.299) (Figure 4A and Table 3). Furthermore, sensitivity analyses supported that there were no significant causal associations of the risk for SLE with circulating omega-3 levels (Figure 4B). No significantly horizontal pleiotropy was found (intercept = 0.004; SE = 0.006, *p* = 0.476) (Figure 4C and Table 3). In addition, the heterogeneity test showed no significant heterogeneity across the selected SNPs (MR-Egger *p* = 0.972 and IVW *p* = 0.975) (Figure 4D and Table 3).

**Table 3:** Causal effects of the risk of SLE on Omega-3.

**Exposure/Outcome**	**Methods**	**SNP (n)**	**Beta**	**95% CI**		***P* value**
SLE/ Omega-3 fatty acids	MR-Egger	41	−0.002	−0.033	0.029	0.876
	IVW	41	0.007	−0.006	0.022	0.299
	Weighted median	41	0.002	−0.019	0.023	0.843
	Weighted mode	41	−0.008	−0.044	0.029	0.688

*Test for Heterogeneity: P = 0.972 (MR-Egger) and P = 0.975 (IVW)*.

*Test for Horizontal pleiotropy: MR-Egger intercept = 0.004, se = 0.006, P = 0.476*.

*OR, odds ratio; IVW, inverse-variance weighted; SLE, systemic lupus erythematosus. The bold values indicate statistical significance with P-value < 0.05*.

**Figure 5:**
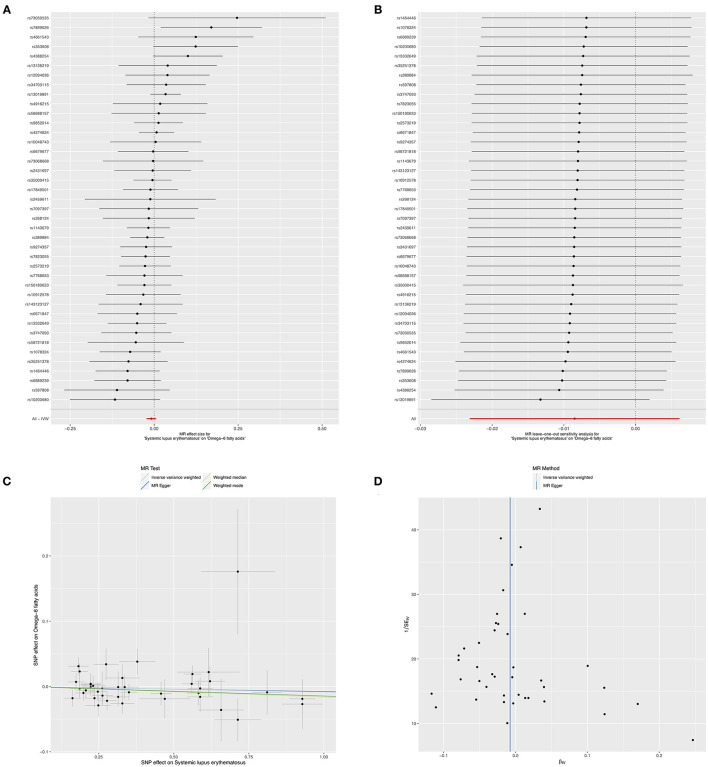
Mendelian randomization study of the effects of SLE on omega-6 levels. Forest plot **(A)**, leave-one-out sensitivity analysis **(B)**, scatter plot **(C)**, and funnel plot **(D)** of the genetically risk of SLE on circulating omega-6 levels.

Moreover, the causality of genetically predicted risk for SLE on the effects of circulating omega-6 levels was investigated, the results implied that there was no evidence of causal links between SLE and omega-6 (IVW method: beta = −0.008, 95% *CI*: −0.023, 0.006, *p* = 0.255; weighted median method: beta = −0.016, 95% *CI*: −0.038, 0.006, *p* = 0.145, respectively) (Figure 5A and Table 4). Furthermore, sensitivity analysis by leaving out each SNP revealed that our results were reliable (Figure 5B). The horizontal pleiotropy test by using MR-Egger method showed a low likelihood of pleiotropy for all of SNPs (intercept = 0.001; SE = 0.006, *p* = 0.931) (Figure 5C and Table 4). Additionally, there was no heterogeneity among all 41 SNPs in the causal effects between SLE and omega-6 (MR-Egger *p* = 0.376 and IVW *p* = 0.419) (Figure 5D and Table 4).

**Table 4:** Causal effects of the risk of SLE on Omega-6.

**Exposure/Outcome**	**Methods**	**SNP (n)**	**Beta**	**95% CI**		***P* value**
SLE/ Omega-6 fatty acids	MR-Egger	41	−0.007	−0.039	0.025	0.659
	IVW	41	−0.008	−0.023	0.006	0.255
	Weighted median	41	−0.016	−0.038	0.006	0.145
	Weighted mode	41	−0.015	−0.043	0.014	0.324

*Test for Heterogeneity: P = 0.376 (MR-Egger) and P = 0.419 (IVW)*.

*Test for Horizontal pleiotropy: MR-Egger intercept = 0.001, se = 0.006, P = 0.931*.

*OR, odds ratio; IVW, inverse-variance weighted; SLE, systemic lupus erythematosus*.

## Discussion

In the current study, the results of MR analysis revealed that the genetically determined per SD increase of circulating omega-3 levels were causally associated with an increased disease risk for SLE. However, the genetic predisposition to omega-6 levels showed no causal relationships with SLE. Moreover, to unveil if there were causal associations of SLE with omega-3 and omega-6 levels, the bidirectional MR analyses were performed, and we found that there were no evidence of causal associations of SLE with omega-3 and omega-6, respectively. It suggested that the genetically determined increase of omega-3 levels may be regarded as a susceptible factor contributing to the pathogenesis of SLE.

Over the past two decades, the associations of omega-3 with human diseases have been documented in several literatures, where omega-3 PUFAs have been demonstrated to be associated with numerous health benefits, such as improvements in cardiovascular health, diabetes, and others (32, 33). In SLE, a number of studies have been conducted to evaluate the effects of supplementation of omega-3 on SLE. Previous clinical studies and meta-analysis reported the potential benefits of daily omega-3 supplementation on the improvements of endothelial function, disease activity, and inflammatory markers in patients with SLE (15, 34–36). However, there are also some controversies about the efficacy of omega-3 supplementation on SLE, where no differences on disease activity of SLE were observed between the omega-3 supplementation and placebo groups (37, 38). The suggestion of benefits reported in previous literatures may be due to the relatively small study population, altered doses of omega-3 supplementation, and follow-up time, these could cause an insufficient power to conclude the associations of omega-3 and SLE. While, our finding revealed that the *OR* (95% *CI*) for SLE was 1.49 (95% *CI*: 1.07, 2.08) per SD increase in circulating omega-3 levels, suggesting that the genetically determined increase of omega-3 levels were positively associated with the disease risk for SLE. In addition, further analysis found that the genetically predicted risk for SLE was not causally linked with the changes of circulating omega-3 levels. Our findings contrasted the results from most previous studies, and such differences might be explained by the following aspects. First, the levels of omega-3 and omega-6 in the current study were defined as circulating metabolites, while omega-3 in previous studies was used as supplements. The supplements of omega-3 could not be equal to the circulating omega-3 levels, and it may cause the contrasting associations with SLE. Second, MR study considered lifetime effects of the SNPs rather than a short period, which could also explain the differences in the results between our study and previous literatures.

Omega-6 PUFAs have been demonstrated to play a crucial role in stimulating the growth of skin and hair, regulating lipid metabolism, and improving bone health (39–41). Additional evidence has highlighted the important roles of omega-6 in cytokine production and monocyte chemotaxis (9, 42). However, as omega-3 and omega-6 compete for the same desaturation and elongation enzymes, the excessive intake of omega-6 causes an increased ratio of omega-6 to omega-3, and competes with benefits of omega-3, increasing the probability of CVD, cancers, and inflammatory diseases (43, 44). Up to date, there were limited studies that have investigated the effects of omega-6 on SLE. Elkan et al. performed a study to explore the associations of dietary habits with subcutaneous adipose tissue (AT) PUFAs in patients with SLE, and they observed that dietary intake of omega-6 (linoleic acid) positively correlated with systemic lupus activity measure (SLAM), and AT omega-6 (arachidonic acid) showed a positive association with the systemic lupus international collaborating clinics (SLICC) damage index (45). Moreover, a prior study has investigated the relationship of the ratio of omega-6 to omega-3 with self-reported disease outcome of SLE, it showed that the increased omega-6 to omega-3 ratio positively correlated with the increase of SLAQ score, but negatively associated with the sleep quality of patients with SLE (17). The findings of our study did not observe the presence of causal links of genetically determined omega-6 with diseased risk for SLE, nor did the causal associations of genetically predicted risk for SLE on the effects of circulating omega-6 levels, suggesting that omega-6 might not be a cause for the onset of SLE.

Our study is also subject to some limitations. First, the genetic data for exposure or outcome are GWAS summary data, which lack the age-specific or sex-specific data, therefore, the MR analyses with age or sex stratification are unavailable. Second, given the varied quality control when conducting individual GWAS, it may produce the potential confounding bias, and make the results that are not easily generalized. Third, the onset or development of SLE are triggered by genetic and several environmental factors, we only evaluated the associations of omega-3 and omega-6 with SLE from a genetic point of view. Finally, both the study population for exposure and outcome came from two independent European ancestries, it might lead to the results that are less generalizable to other ancestry.

Despite these limitations, the present study also has some advantages. We used genetic summary statistics from two large-scale GWASs that were the most representative studies with large-scale genome-wide association data sources in European ancestry, thus enhancing the statistical power for MR analysis. In addition, as compared with the traditional observational study, the use of MR could minimize the residual confounding and avoid reverse causation bias. Furthermore, there was no or very limited overlap in the study population between exposure and outcome datasets, thus it would be more effective to control the type 1 error at a low level.

## Conclusions

In summary, using the approach of MR analysis, we observed a positively causal link between omega-3 and SLE, but no causal association of omega-6 with SLE was revealed. Our findings provided the evidence of that omega-3 might increase the risk for SLE, which would be insightful for the current dietary fish oil supplements. However, due to the study limitation, further large-scale studies or longitudinal studies are required to validate this finding.

## Data Availability Statement

The original contributions presented in the study are included in the article/Supplementary Material, further inquiries can be directed to the corresponding authors.

## Author Contributions

H-FP and JN conceived the presented idea. PW and KX developed the theory and performed the computations. Y-YX and Y-SH verified the analytical methods. PW and Y-QH drafted the manuscript. All authors discussed the results and contributed to the final manuscript.

## Funding

This study was funded by grants from National Natural Science Foundation of China (Nos. 81872687 and 82103932), Anhui Provincial Natural Science Foundation (Nos. 2108085Y26 and 2108085QH361), Research Fund of Anhui Institute of Translational Medicine (No. 2021zhyx-B04), and Nature Science Foundation of Anhui Medical University (No. 2020xkj0).

## Conflict of Interest

The authors declare that the research was conducted in the absence of any commercial or financial relationships that could be construed as a potential conflict of interest.

## Publisher's Note

All claims expressed in this article are solely those of the authors and do not necessarily represent those of their affiliated organizations, or those of the publisher, the editors and the reviewers. Any product that may be evaluated in this article, or claim that may be made by its manufacturer, is not guaranteed or endorsed by the publisher.

## References

[1] Lisnevskaia L Murphy G Isenberg D. Systemic lupus erythematosus. Lancet. (2014) 384:1878–88. 10.1016/S0140-6736(14)60128-8 24881804

[2] Kaul A Gordon C Crow MK Touma Z Urowitz MB van Vollenhoven R. Systemic lupus erythematosus. Nat Rev Dis Primers. (2016) 2:16039. 10.1038/nrdp.2016.39 27306639

[3] Barbhaiya M Costenbader KH. Environmental exposures and the development of systemic lupus erythematosus. Curr Opin Rheumatol. (2016) 28:497–505. 10.1097/BOR.0000000000000318 27428889 PMC4965307

[4] Leffers HCB Lange T Collins C Ulff-Moller CJ Jacobsen S. The study of interactions between genome and exposome in the development of systemic lupus erythematosus. Autoimmun Rev. (2019) 18:382–92. 10.1016/j.autrev.2018.11.005 30772495

[5] Teruel M Alarcon-Riquelme ME. The genetic basis of systemic lupus erythematosus: What are the risk factors and what have we learned. J Autoimmun. (2016) 74:161–75. 10.1016/j.jaut.2016.08.001 27522116

[6] Gorji AE Roudbari Z Alizadeh A Sadeghi B. Investigation of systemic lupus erythematosus (SLE) with integrating transcriptomics and genome wide association information. Gene. (2019) 706:181–7. 10.1016/j.gene.2019.05.004 31082500

[7] Owen KA Price A Ainsworth H Aidukaitis BN Bachali P Catalina MD. Analysis of Trans-Ancestral SLE Risk Loci Identifies Unique Biologic Networks and Drug Targets in African and European Ancestries. Am J Hum Genet. (2020) 107:864–81. 10.1016/j.ajhg.2020.09.007 33031749 PMC7675009

[8] James MJ Gibson RA Cleland LG. Dietary polyunsaturated fatty acids and inflammatory mediator production. Am J Clin Nutr. (2000) 71(1 Suppl):343S-8S. 10.1093/ajcn/71.1.343s 10617994

[9] Calder PC. Omega-3 fatty acids and inflammatory processes: from molecules to man. Biochem Soc Trans. (2017) 45:1105–15. 10.1042/BST20160474 28900017

[10] D'Angelo S Motti ML Meccariello R. omega-3 and omega-6 Polyunsaturated Fatty Acids, Obesity and Cancer. Nutrients. (2020) 12:2751. 10.3390/nu12092751 32927614 PMC7551151

[11] Derbyshire E. Oily Fish and Omega-3s across the life stages: a focus on intakes and future directions. Front Nutr. (2019) 6:165. 10.3389/fnut.2019.00165 31781570 PMC6861329

[12] Galarraga B Ho M Youssef HM Hill A McMahon H Hall C. Cod liver oil (n-3 fatty acids) as an non-steroidal anti-inflammatory drug sparing agent in rheumatoid arthritis. Rheumatology (Oxford). (2008) 47:665–9. 10.1093/rheumatology/ken024 18362100

[13] Li X Bi X Wang S Zhang Z Li F Zhao AZ. Therapeutic Potential of omega-3 polyunsaturated fatty acids in human autoimmune diseases. Front Immunol. (2019) 10:2241. 10.3389/fimmu.2019.02241 31611873 PMC6776881

[14] Goldberg RJ Katz J. A meta-analysis of the analgesic effects of omega-3 polyunsaturated fatty acid supplementation for inflammatory joint pain. Pain. (2007) 129:210–23. 10.1016/j.pain.2007.01.020 17335973

[15] Duarte-Garcia A Myasoedova E Karmacharya P Hocaoglu M Murad MH Warrington KJ. Effect of omega-3 fatty acids on systemic lupus erythematosus disease activity: a systematic review and meta-analysis. Autoimmun Rev. (2020) 19:102688. 10.1016/j.autrev.2020.102688 33131703

[16] Pestka JJ Vines LL Bates MA He K Langohr I. Comparative effects of n-3, n-6 and n-9 unsaturated fatty acid-rich diet consumption on lupus nephritis, autoantibody production and CD4+ T cell-related gene responses in the autoimmune NZBWF1 mouse. PLoS ONE. (2014) 9:e100255. 10.1371/journal.pone.0100255 24945254 PMC4063768

[17] Charoenwoodhipong P Harlow SD Marder W Hassett AL McCune WJ Gordon C. Dietary omega polyunsaturated fatty acid intake and patient-reported outcomes in systemic lupus erythematosus: the michigan lupus epidemiology and surveillance program. Arthritis Care Res (Hoboken). (2020) 72:874–81. 10.1002/acr.23925 31074595 PMC6842394

[18] Bennett DA Holmes MV. Mendelian randomisation in cardiovascular research: an introduction for clinicians. Heart. (2017) 103:1400–7. 10.1136/heartjnl-2016-310605 28596306 PMC5574403

[19] Liao LZ Zhang SZ Li WD Liu Y Li JP Zhuang XD. Serum albumin and atrial fibrillation: insights from epidemiological and mendelian randomization studies. Eur J Epidemiol. (2020) 35:113–22. 10.1007/s10654-019-00583-6 31741136

[20] Scheller Madrid A Rasmussen KL Rode L Frikke-Schmidt R Nordestgaard BG Bojesen SE. Observational and genetic studies of short telomeres and Alzheimer's disease in 67,000 and 152,000 individuals: a Mendelian randomization study. Eur J Epidemiol. (2020) 35:147–56. 10.1007/s10654-019-00563-w 31564046

[21] Wootton RE Greenstone HSR Abdellaoui A Denys D Verweij KJH Munafo MR. Bidirectional effects between loneliness, smoking and alcohol use: evidence from a Mendelian randomization study. Addiction. (2020). 10.1101/19006767 32542815

[22] Wang P Liu L Lei SF. Causal effects of homocysteine levels on the changes of bone mineral density and risk for bone fracture: a two-sample mendelian randomization study. Clin Nutr. (2021) 40:1588–95. 10.1016/j.clnu.2021.02.045 33744603

[23] Evans DM Davey Smith G. Mendelian randomization: new applications in the coming age of hypothesis-free causality. Annu Rev Genomics Hum Genet. (2015) 16:327–50. 10.1146/annurev-genom-090314-050016 25939054

[24] Pierce BL Burgess S. Efficient design for Mendelian randomization studies: subsample and 2-sample instrumental variable estimators. Am J Epidemiol. (2013) 178:1177–84. 10.1093/aje/kwt084 23863760 PMC3783091

[25] Hemani G Zheng J Elsworth B Wade KH Haberland V Baird D. The MR-Base platform supports systematic causal inference across the human phenome. Elife. (2018) 7:e34408. 10.7554/eLife.34408 29846171 PMC5976434

[26] Bowden J Davey Smith G Burgess S. Mendelian randomization with invalid instruments: effect estimation and bias detection through Egger regression. Int J Epidemiol. (2015) 44:512–25. 10.1093/ije/dyv080 26050253 PMC4469799

[27] Kettunen J Demirkan A Wurtz P Draisma HH Haller T Rawal R. Genome-wide study for circulating metabolites identifies 62 loci and reveals novel systemic effects of LPA. Nat Commun. (2016) 7:11122. 10.1038/ncomms11122 27005778 PMC4814583

[28] Bentham J Morris DL Graham DSC Pinder CL Tombleson P Behrens TW. Genetic association analyses implicate aberrant regulation of innate and adaptive immunity genes in the pathogenesis of systemic lupus erythematosus. Nat Genet. (2015) 47:1457–64. 10.1038/ng.3434 26502338 PMC4668589

[29] Pagoni P Dimou NL Murphy N Stergiakouli E. Using Mendelian randomisation to assess causality in observational studies. Evid Based Ment Health. (2019) 22:67–71. 10.1136/ebmental-2019-300085 30979719 PMC10270458

[30] Bowden J Davey Smith G Haycock PC Burgess S. Consistent estimation in mendelian randomization with some invalid instruments using a weighted median estimator. Genet Epidemiol. (2016) 40:304–14. 10.1002/gepi.21965 27061298 PMC4849733

[31] Burgess S Thompson SG. Interpreting findings from Mendelian randomization using the MR-Egger method. Eur J Epidemiol. (2017) 32:377–89. 10.1007/s10654-017-0255-x 28527048 PMC5506233

[32] Shahidi F Ambigaipalan P. Omega-3 polyunsaturated fatty acids and their health benefits. Annu Rev Food Sci Technol. (2018) 9:345–81. 10.1146/annurev-food-111317-095850 29350557

[33] Elagizi A Lavie CJ O'Keefe E Marshall K O'Keefe JH Milani RV. An Update on Omega-3 polyunsaturated fatty acids and cardiovascular health. Nutrients. (2021) 13:204. 10.3390/nu13010204 33445534 PMC7827286

[34] Das UN. Beneficial effect of eicosapentaenoic and docosahexaenoic acids in the management of systemic lupus erythematosus and its relationship to the cytokine network. Prostaglandins Leukot Essent Fatty Acids. (1994) 51:207–13. 10.1016/0952-3278(94)90136-8 7824535

[35] Duffy EM Meenagh GK McMillan SA Strain JJ Hannigan BM Bell AL. The clinical effect of dietary supplementation with omega-3 fish oils and/or copper in systemic lupus erythematosus. J Rheumatol. (2004) 31:1551–6. 15290734

[36] Arriens C Hynan LS Lerman RH Karp DR Mohan C. Placebo-controlled randomized clinical trial of fish oil's impact on fatigue, quality of life, and disease activity in Systemic Lupus Erythematosus. Nutr J. (2015) 14:82. 10.1186/s12937-015-0068-2 26283629 PMC4538741

[37] Bello KJ Fang H Fazeli P Bolad W Corretti M Magder LS. Omega-3 in SLE: a double-blind, placebo-controlled randomized clinical trial of endothelial dysfunction and disease activity in systemic lupus erythematosus. Rheumatol Int. (2013) 33:2789–96. 10.1007/s00296-013-2811-3 23817872 PMC3805738

[38] Clark WF Parbtani A Huff MW Reid B Holub BJ Falardeau P. Omega-3 fatty acid dietary supplementation in systemic lupus erythematosus. Kidney Int. (1989) 36:653–60. 10.1038/ki.1989.242 2811063

[39] Hooper L Al-Khudairy L Abdelhamid AS Rees K Brainard JS Brown TJ. Omega-6 fats for the primary and secondary prevention of cardiovascular disease. Cochrane Database Syst Rev. (2018) 7:CD011094. 10.1002/14651858.CD011094.pub3 30019765 PMC6513455

[40] Le Floc'h C Cheniti A Connetable S Piccardi N Vincenzi C Tosti A. Effect of a nutritional supplement on hair loss in women. J Cosmet Dermatol. (2015) 14:76–82. 10.1111/jocd.12127 25573272

[41] Bjorklund G Dadar M Dosa MD Chirumbolo S Pen JJ. Insights on dietary omega-6/omega-3 polyunsaturated fatty acid (PUFA) ratio in oxidative metabolic pathways of oncological bone disease and global health. Curr Med Chem. (2020) 28:1672–82. 10.2174/0929867327666200427095331 32338204

[42] Melody SM Vincent R Mori TA Mas E Barden AE Waddell BJ. Effects of omega-3 and omega-6 fatty acids on human placental cytokine production. Placenta. (2015) 36:34–40. 10.1016/j.placenta.2014.10.013 25468541

[43] Simopoulos AP. The importance of the ratio of omega-6/omega-3 essential fatty acids. Biomed Pharmacother. (2002) 56:365–79. 10.1016/S0753-3322(02)00253-6 12442909

[44] Simopoulos AP. Evolutionary aspects of diet, the omega-6/omega-3 ratio and genetic variation: nutritional implications for chronic diseases. Biomed Pharmacother. (2006) 60:502–7. 10.1016/j.biopha.2006.07.080 17045449

[45] Elkan AC Anania C Gustafsson T Jogestrand T Hafstrom I Frostegard J. Diet and fatty acid pattern among patients with SLE: associations with disease activity, blood lipids and atherosclerosis. Lupus. (2012) 21:1405–11. 10.1177/0961203312458471 22930204

