# Supplementary material for: Genetically Predicted Circulating Omega-3 Fatty Acids Levels Are Causally Associated With Increased Risk for Systemic Lupus Erythematosus
Source: Front Nutr. 2022 Feb 9;9:783338. doi: 10.3389/fnut.2022.783338 (PMC8864316; doi:10.3389/fnut.2022.783338)
Supplement: Supplementary file 1 [file Table_1.DOCX]

**Supplementary Table 1 Genome-wide significant variants on Omega-3 and their association with SLE**

| SNP | Chr | Position | Effect allele | Other allele | EAF | Omega-3 | | | |  | SLE | | | |
| --- | --- | --- | --- | --- | --- | --- | --- | --- | --- | --- | --- | --- | --- | --- |
|  |  |  |  |  |  | n | Beta | SE | *P* |  | n | Beta | SE | *P* |
| rs1077835 | 15 | 58723426 | G | A | 0.250 | 13538 | 0.089 | 0.014 | 1.08E-09 |  | 14267 | 0.086 | 0.034 | 0.051 |
| rs11604424 | 11 | 116651115 | T | C | 0.756 | 13540 | -0.090 | 0.014 | 3.32E-10 |  | 14267 | 0.030 | 0.034 | 0.376 |
| rs1260326 | 2 | 27730940 | C | T | 0.637 | 13544 | -0.097 | 0.013 | 3.37E-14 |  | 14267 | -0.049 | 0.029 | 0.091 |
| rs143988316 | 19 | 19667254 | T | C | 0.069 | 13539 | -0.171 | 0.024 | 2.95E-12 |  | 14267 | -0.030 | 0.057 | 0.596 |
| rs174546 | 11 | 61569830 | T | C | 0.403 | 13544 | -0.154 | 0.012 | 1.19E-34 |  | 14267 | -0.073 | 0.032 | 0.064 |

EAF: effect allele frequency; SE: standard error; SLE: Systemic lupus erythematosus
