# Supplementary material for: Genetically Predicted Circulating Omega-3 Fatty Acids Levels Are Causally Associated With Increased Risk for Systemic Lupus Erythematosus
Source: Front Nutr. 2022 Feb 9;9:783338. doi: 10.3389/fnut.2022.783338 (PMC8864316; doi:10.3389/fnut.2022.783338)
Supplement: Supplementary file 2 [file Table_2.DOCX]

**Supplementary Table 2 Genome-wide significant variants on Omega-6 fatty acids and their association with SLE**

| SNP | Chr | Position | Effect allele | Other allele | EAF | Omega-6 | | | |  | SLE | | | |
| --- | --- | --- | --- | --- | --- | --- | --- | --- | --- | --- | --- | --- | --- | --- |
|  |  |  |  |  |  | n | Beta | SE | *P* |  | n | Beta | SE | *P* |
| rs11591147 | 1 | 55505647 | T | G | 0.029 | 13502 | -0.309 | 0.040 | 1.12E-14 |  | 14267 | -0.342 | 0.120 | 0.064 |
| rs1260326 | 2 | 27730940 | C | T | 0.637 | 13506 | -0.078 | 0.013 | 9.73E-10 |  | 14267 | -0.049 | 0.029 | 0.091 |
| rs144064722 | 4 | 73406173 | G | A | 0.026 | 13500 | 0.237 | 0.040 | 2.29E-09 |  | 14267 | 0.039 | 0.077 | 0.611 |
| rs174418 | 15 | 58687603 | C | T | 0.562 | 13504 | -0.098 | 0.013 | 5.99E-15 |  | 14267 | -0.010 | 0.056 | 0.858 |
| rs1800588 | 15 | 58723675 | T | C | 0.249 | 13504 | 0.143 | 0.014 | 9.47E-23 |  | 14267 | 0.086 | 0.033 | 0.068 |
| rs3741298 | 11 | 116657561 | T | C | 0.770 | 13503 | -0.143 | 0.015 | 7.32E-23 |  | 14267 | 0.041 | 0.040 | 0.308 |
| rs7412 | 19 | 45412079 | T | C | 0.057 | 13501 | -0.272 | 0.028 | 8.06E-22 |  | 14267 | 0.113 | 0.054 | 0.037 |
| rs79225634 | 5 | 74619639 | T | C | 0.352 | 13503 | 0.085 | 0.013 | 7.52E-11 |  | 14267 | 0.020 | 0.032 | 0.532 |
| rs821840 | 16 | 56993886 | G | A | 0.252 | 13503 | 0.086 | 0.015 | 4.77E-09 |  | 14267 | -0.094 | 0.035 | 0.087 |

EAF: effect allele frequency; SE: standard error; SLE: Systemic lupus erythematosus
