# Supplementary material for: Genetically Predicted Circulating Omega-3 Fatty Acids Levels Are Causally Associated With Increased Risk for Systemic Lupus Erythematosus
Source: Front Nutr. 2022 Feb 9;9:783338. doi: 10.3389/fnut.2022.783338 (PMC8864316; doi:10.3389/fnut.2022.783338)
Supplement: Supplementary file 4 [file Table_4.DOCX]

**Supplementary Table 4 Genome-wide significant variants on the risk for SLE and their association with Omega-6**

| SNP | Chr | Position | Effect allele | Other allele | EAF | SLE | | | |  | Omega-6 | | | |
| --- | --- | --- | --- | --- | --- | --- | --- | --- | --- | --- | --- | --- | --- | --- |
|  |  |  |  |  |  | n | Beta | SE | *P* |  | n | Beta | SE | *P* |
| rs10048743 | 2 | 213890232 | G | T | 0.141 | 14267 | -0.231 | 0.041 | 2.04E-08 |  | 13502 | -0.001 | 0.016 | 0.932 |
| rs10200680 | 2 | 223961877 | C | T | 0.855 | 14267 | -0.248 | 0.042 | 4.96E-09 |  | 13501 | 0.029 | 0.017 | 0.086 |
| rs1078324 | 5 | 149202268 | A | C | 0.049 | 14267 | -0.713 | 0.078 | 7.11E-20 |  | 13502 | 0.051 | 0.033 | 0.124 |
| rs10912578 | 1 | 173251856 | A | G | 0.301 | 14267 | -0.247 | 0.031 | 1.65E-15 |  | 13500 | 0.008 | 0.014 | 0.572 |
| rs1143679 | 16 | 31276811 | A | G | 0.131 | 14267 | 0.582 | 0.040 | 5.03E-48 |  | 13502 | -0.010 | 0.019 | 0.598 |
| rs12094036 | 1 | 183558174 | C | T | 0.081 | 14267 | -0.329 | 0.058 | 1.37E-08 |  | 13499 | -0.013 | 0.021 | 0.526 |
| rs13019891 | 2 | 113829869 | G | T | 0.548 | 14267 | -0.562 | 0.029 | 1.65E-83 |  | 13506 | -0.019 | 0.013 | 0.123 |
| rs13136219 | 4 | 102743687 | C | T | 0.620 | 14267 | -0.174 | 0.028 | 3.50E-10 |  | 13501 | -0.007 | 0.013 | 0.601 |
| rs13332649 | 16 | 85966683 | A | G | 0.802 | 14267 | -0.315 | 0.038 | 5.43E-17 |  | 13502 | 0.016 | 0.014 | 0.245 |
| rs143123127 | 17 | 38007190 | A | G | 0.030 | 14267 | 0.470 | 0.084 | 2.23E-08 |  | 13502 | -0.019 | 0.030 | 0.515 |
| rs1464446 | 3 | 146601295 | T | G | 0.178 | 14267 | -0.329 | 0.040 | 2.79E-16 |  | 13505 | 0.026 | 0.016 | 0.114 |
| rs150180633 | 6 | 31010047 | C | T | 0.983 | 14267 | 0.928 | 0.069 | 2.66E-41 |  | 12801 | -0.027 | 0.038 | 0.475 |
| rs17849501 | 1 | 183542323 | C | T | 0.940 | 14267 | 0.811 | 0.050 | 1.81E-59 |  | 13502 | -0.009 | 0.034 | 0.801 |
| rs2431697 | 5 | 159879978 | C | T | 0.431 | 14267 | -0.223 | 0.029 | 2.60E-14 |  | 13506 | 0.001 | 0.013 | 0.928 |
| rs2459611 | 2 | 191939187 | C | T | 0.125 | 14267 | 0.261 | 0.045 | 7.62E-09 |  | 13506 | -0.003 | 0.026 | 0.895 |
| rs2573219 | 2 | 233288667 | C | A | 0.086 | 14267 | 0.588 | 0.043 | 1.13E-42 |  | 13505 | -0.016 | 0.023 | 0.469 |
| rs268124 | 2 | 65654364 | C | T | 0.274 | 14267 | 0.186 | 0.032 | 8.60E-09 |  | 13503 | -0.003 | 0.013 | 0.817 |
| rs34703115 | 2 | 40282854 | C | T | 0.032 | 14267 | -0.616 | 0.105 | 4.08E-09 |  | 13504 | -0.022 | 0.037 | 0.547 |
| rs35000415 | 7 | 128585616 | C | T | 0.899 | 14267 | 0.588 | 0.042 | 1.86E-45 |  | 13504 | -0.003 | 0.017 | 0.848 |
| rs35251378 | 19 | 10459969 | A | G | 0.269 | 14267 | -0.236 | 0.032 | 3.61E-13 |  | 13501 | 0.018 | 0.014 | 0.213 |
| rs353608 | 11 | 35101738 | G | A | 0.547 | 14267 | 0.186 | 0.028 | 2.93E-11 |  | 13505 | 0.023 | 0.012 | 0.062 |
| rs3747093 | 22 | 21984379 | A | G | 0.201 | 14267 | 0.262 | 0.035 | 2.88E-14 |  | 13502 | -0.014 | 0.014 | 0.296 |
| rs389884 | 6 | 31940897 | G | A | 0.073 | 14267 | 0.928 | 0.043 | 2.92E-102 |  | 11659 | -0.019 | 0.024 | 0.444 |
| rs4274624 | 2 | 191958656 | C | T | 0.231 | 14267 | -0.560 | 0.033 | 9.73E-66 |  | 13504 | -0.004 | 0.015 | 0.783 |
| rs4388254 | 5 | 133428601 | C | T | 0.929 | 14267 | 0.378 | 0.060 | 3.71E-10 |  | 13503 | 0.038 | 0.020 | 0.056 |
| rs4661543 | 1 | 15229101 | G | T | 0.872 | 14267 | 0.274 | 0.042 | 9.40E-11 |  | 13505 | 0.034 | 0.024 | 0.160 |
| rs4916215 | 1 | 173314540 | C | T | 0.254 | 14267 | 0.223 | 0.034 | 5.07E-11 |  | 13503 | 0.004 | 0.016 | 0.799 |
| rs58688157 | 11 | 625085 | A | G | 0.731 | 14267 | -0.223 | 0.034 | 2.97E-11 |  | 13501 | -0.003 | 0.016 | 0.871 |
| rs58721818 | 6 | 138243739 | C | T | 0.975 | 14267 | 0.658 | 0.076 | 3.38E-18 |  | 13502 | -0.036 | 0.048 | 0.453 |
| rs597808 | 12 | 111973358 | G | A | 0.533 | 14267 | -0.163 | 0.029 | 3.51E-08 |  | 13504 | 0.018 | 0.013 | 0.149 |
| rs6671847 | 1 | 161478810 | A | G | 0.487 | 14267 | 0.199 | 0.029 | 6.64E-12 |  | 13504 | -0.010 | 0.012 | 0.425 |
| rs6679677 | 1 | 114303808 | A | C | 0.091 | 14267 | 0.336 | 0.046 | 4.55E-13 |  | 13504 | -0.001 | 0.018 | 0.942 |
| rs6889239 | 5 | 150457771 | C | T | 0.257 | 14267 | 0.278 | 0.032 | 2.19E-18 |  | 13501 | -0.022 | 0.014 | 0.117 |
| rs7097397 | 10 | 50025396 | A | G | 0.395 | 14267 | -0.186 | 0.029 | 8.60E-11 |  | 12135 | 0.003 | 0.014 | 0.806 |
| rs73050535 | 12 | 5012503 | C | T | 0.970 | 14267 | -0.713 | 0.124 | 9.11E-09 |  | 12475 | -0.176 | 0.096 | 0.068 |
| rs73068668 | 19 | 55763262 | A | G | 0.095 | 14267 | -0.315 | 0.057 | 4.40E-08 |  | 13501 | 0.001 | 0.024 | 0.960 |
| rs7768653 | 6 | 106574794 | C | T | 0.401 | 14267 | -0.207 | 0.030 | 3.11E-12 |  | 13504 | 0.006 | 0.012 | 0.634 |
| rs7823055 | 8 | 55511676 | G | T | 0.423 | 14267 | -0.351 | 0.029 | 1.64E-34 |  | 13504 | 0.009 | 0.013 | 0.509 |
| rs7899626 | 10 | 63825561 | C | T | 0.637 | 14267 | 0.182 | 0.033 | 4.19E-08 |  | 12135 | 0.031 | 0.014 | 0.025 |
| rs9274357 | 6 | 32632457 | C | G | 0.776 | 14267 | 0.457 | 0.035 | 1.28E-38 |  | 13504 | -0.011 | 0.018 | 0.546 |
| rs9852014 | 3 | 129084581 | G | A | 0.925 | 14267 | 0.621 | 0.049 | 2.26E-36 |  | 13503 | 0.008 | 0.023 | 0.723 |

EAF: effect allele frequency; SE: standard error; SLE: Systemic lupus erythematosus;
